# Persistent Patellar Dislocation Following Correction of a Lower Limb Length Discrepancy: A Case Report

**DOI:** 10.7759/cureus.97882

**Published:** 2025-11-26

**Authors:** Hugo Santos, Francisco Guerra Pinto, Rui Domingos, Gonçalo Rodrigues, Dúnio Jácome Pacheco, Estanqueiro Guarda

**Affiliations:** 1 Orthopaedics and Trauma, Hospital Ortopédico de Sant’Ana, Lisbon, PRT; 2 Orthopaedic Surgery, Hospital Ortopédico de Sant’Ana, Lisbon, PRT

**Keywords:** case report, femoropatellar instability, lengthening, lower limb length discrepancy, trochleoplasty

## Abstract

Lower limb length discrepancy is common, but only a small proportion of cases are clinically significant. Distraction osteogenesis allows correction at any age without reducing final height, though long-term biomechanical consequences may arise. We report the case of a young patient who underwent femoral lengthening for a 4.5 cm discrepancy and, five years later, developed chronic patellar instability with persistent dislocation. Imaging revealed trochlear dysplasia, lateralization of the tibial tuberosity, medial patellofemoral ligament (MPFL) insufficiency, and increased quadriceps tension. A combined surgical approach was performed, including open trochleoplasty, distalization and medialization osteotomy of the tibial tuberosity, MPFL reconstruction, and quadriceps lengthening using a V-Y technique. At one-year follow-up, the patient achieved a pain-free range of motion of 0°-85°, with no recurrent instability and Lysholm and Kujala scores of 71 and 65, respectively. This case illustrates the importance of long-term follow-up after lengthening procedures and highlights the effectiveness of tailored, multimodal surgery in managing complex patellar instability.

## Introduction

Lower limb length discrepancy is a very common condition; however, only 1 in every 1000 people presents clinically significant discrepancies - that is, greater than 2 cm. The etiology may be due to congenital defects, physeal injuries, or pathologies such as cerebral palsy or poliomyelitis [1].

When surgical correction is indicated, two alternative strategies may be employed: either growth modulation by epiphysiodesis, which consists of inducing partial or complete closure of the growth plate in the longer limb to allow the shorter side to “catch up,” or distraction osteogenesis, a technique based on the principle of gradually distracting the callus formed after a subperiosteal osteotomy of a long bone. This controlled, progressive separation stimulates new bone formation within the distraction gap, allowing regeneration of living bone with comparable structure, strength, and width to native bone. Furthermore, peripheral nerves, vessels, muscles, tendons, ligaments, and skin adapt gradually in proportion to the lengthened segment, thereby preserving limb function and final stature [2-4].

While these methods effectively correct limb-length inequality, few studies have addressed their long-term biomechanical impact on adjacent joints, particularly the knee extensor mechanism. During femoral lengthening, progressive tension within the quadriceps and surrounding soft tissues can lead to stiffness, contracture, or even altered patellofemoral tracking. Persistent quadriceps tightness may compromise knee flexion and predispose to patellar maltracking or instability, particularly in patients with underlying condylar hypoplasia or valgus alignment. These complications have been described in the pediatric population, where contracture of the extensor mechanism after lengthening occasionally requires surgical release or quadricepsplasty to restore mobility and correct patellar position. In one series by Martin et al., 79 femoral lengthenings exceeding 2 cm were reviewed, of which seven required re-intervention, two due to obligate patellar dislocation [5]. 

In the case we report, a young patient underwent femoral lengthening to correct a 4.5 cm discrepancy and, five years later, developed chronic femoropatellar instability with persistent dislocation. Patellar instability has a multifactorial etiology, arising from an imbalance between static and dynamic stabilizers, and nearly 60% of patients present more than one anatomical alteration [6]. In this patient, almost all major predisposing factors were present, including trochlear dysplasia, tibial tuberosity malalignment, medial patellofemoral ligament (MPFL) insufficiency, and quadriceps tension, and their combined effect was further exacerbated by the prior femoral lengthening procedure. The sequential occurrence of complications and accumulation of risk factors make this case particularly illustrative of how altered limb biomechanics can culminate in chronic instability. Computed tomography (CT) or magnetic resonance imaging (MRI) is fundamental in identifying these abnormalities and guiding a patient-specific surgical plan [7].

## Case presentation

Ethical approval for this work was granted by the institutional ethics committee. Written informed consent was obtained from the patient prior to publication, including consent for the use of clinical images and radiographs. All identifying details have been removed or anonymized to preserve patient confidentiality in accordance with the Declaration of Helsinki.

**Figure 1:**
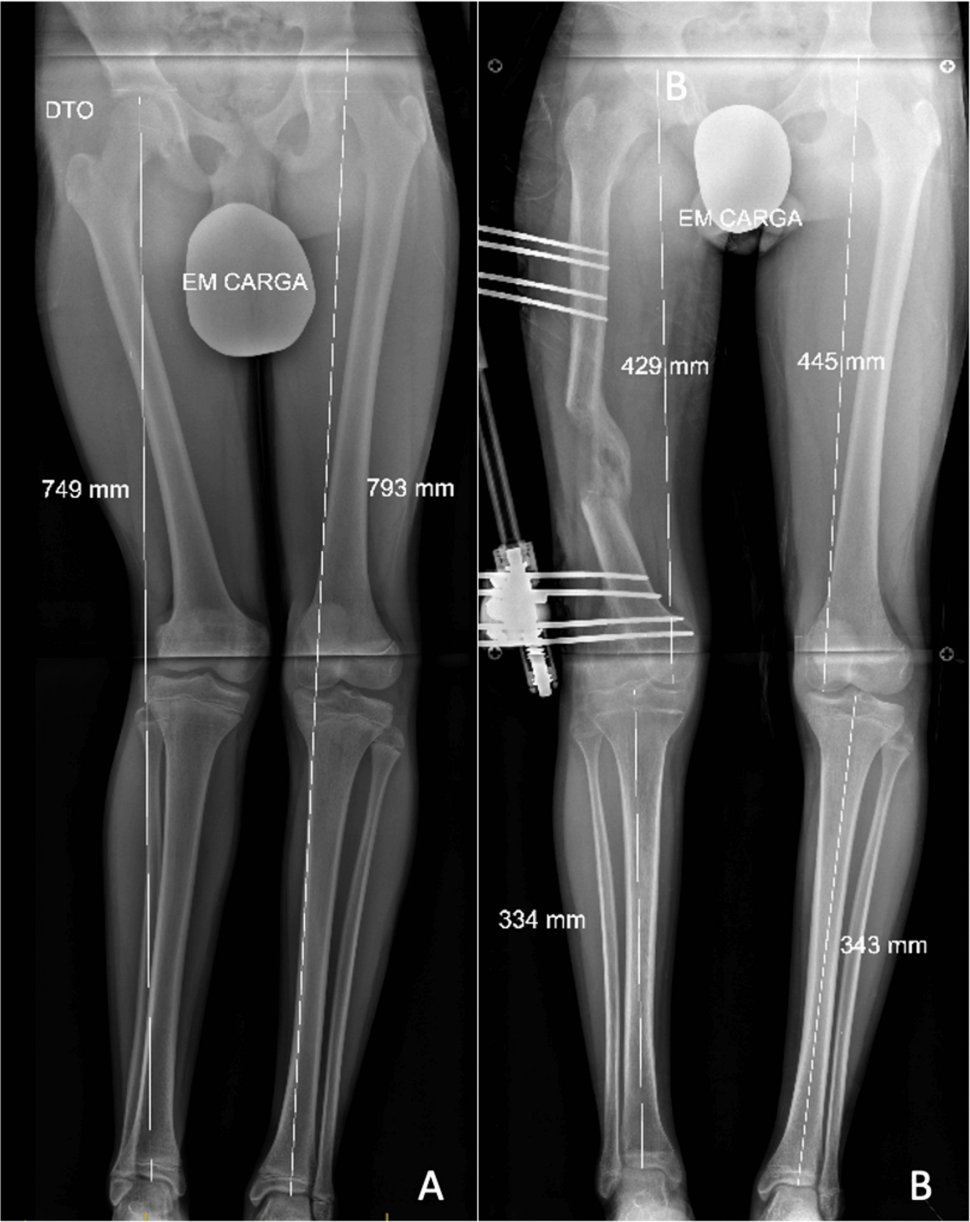
Radiographic result of the lengthening (A) Preoperative radiograph showing a 4.5 cm right femoral length discrepancy; (B) post-distraction radiograph showing correction at the time of lengthening arrest, although a residual discrepancy of 15 mm remained.

This patient had been followed at our institution since the age of 14, when he first presented with a 4.5 cm right lower limb discrepancy and a 12° valgus deformity of the knee (Figure 1A). He denied any history of trauma or infection that might have prompted earlier medical consultation. The case was therefore interpreted as a probable congenital short femur type 1A, according to Paley’s classification, diagnosed at a late stage. At the age of 15, the patient underwent limb lengthening with corticotomy and placement of a monoplane external fixator. Distraction was planned at 1 mm/day, initiated one week after surgery, and continued for 117 days, prolonged due to difficulties in compliance and adaptation to the device. The fixator was maintained for an additional three months to allow regenerative consolidation. Correction was partially successful: the initial discrepancy of 45 mm was reduced to 15 mm, with partial correction of valgus, though a residual deformity of the regenerate relative to the femoral anatomical axis remained (Figure 1B).

**Figure 2:**
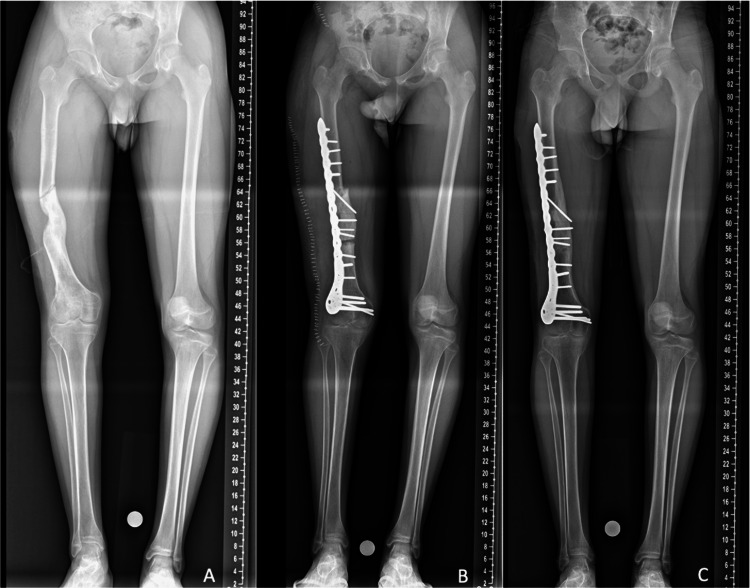
Pre- and postoperative radiographs illustrating the femoral fracture and the result after osteosynthesis (A) Diaphyseal femoral fracture at the regenerate-diaphysis junction; (B) immediate postoperative long-standing radiograph showing correction of alignment and fracture fixation; (C) six-month follow-up radiograph demonstrating consolidation.

One month after removal of the fixator, the patient sustained a fall resulting in a diaphyseal fracture of the right femur (Figure 2A). Fracture fixation was performed while simultaneously correcting the residual deformity by means of a double osteotomy and interposition of two autologous iliac crest bone-graft wedges, stabilized with a plate (Figure 2B). The postoperative course was uneventful, and the implant was removed after 18 months.

**Figure 3:**
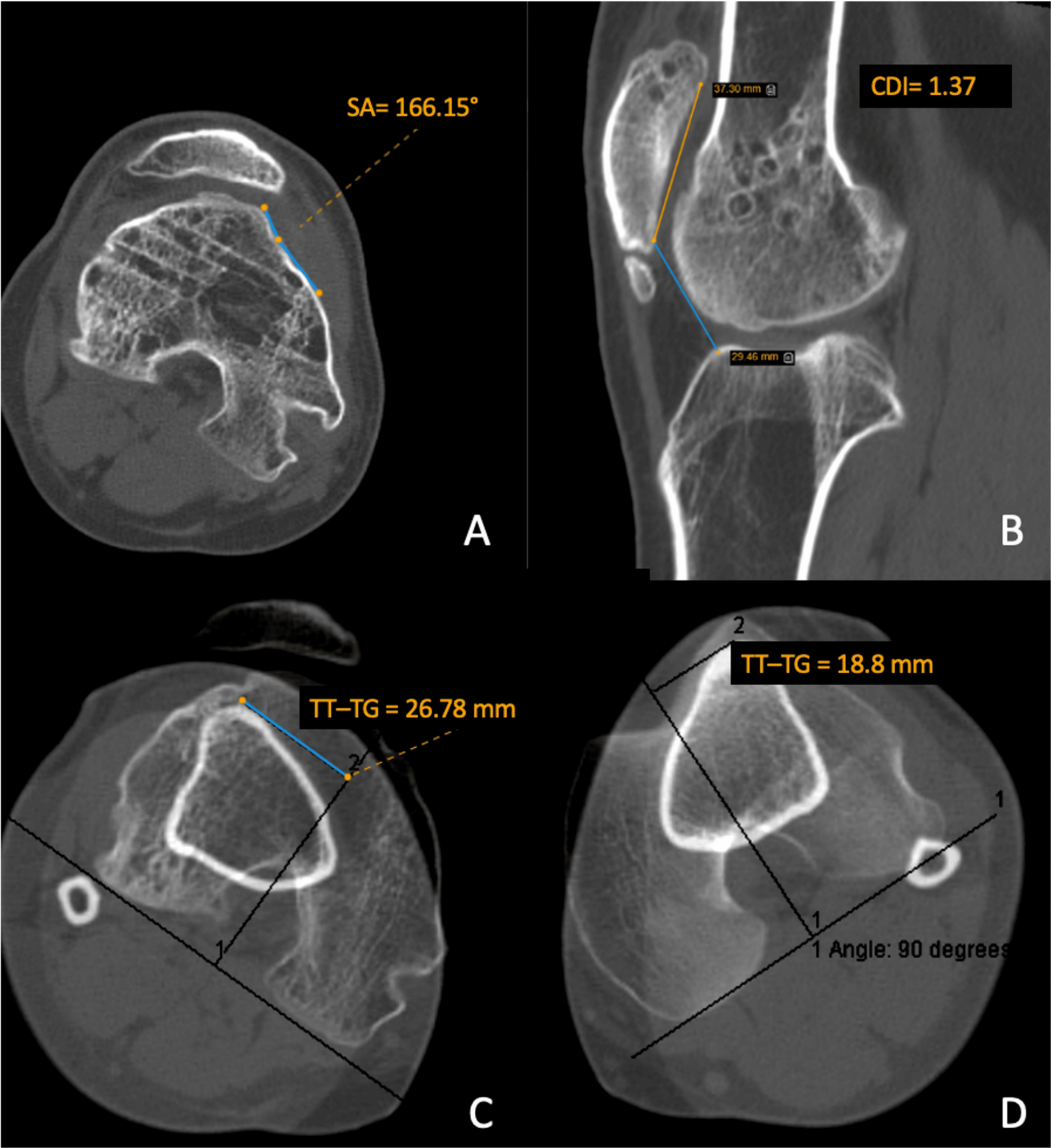
Preoperative CT assessment of patellofemoral parameters (A) Axial CT image at the trochlear groove showing sulcus angle (SA) = 166°; (B) sagittal CT image showing Caton-Deschamps index (CDI) = 1.37; (C) axial CT image showing tibial tubercle-trochlear groove (TT-TG) distance = 26.8 mm; (D) contralateral TT-TG distance = 18.8 mm.

Five years later, the patient returned with progressive limitation of knee mobility. On examination, he had a permanent lateral patellar dislocation of the right knee and a range of motion of 0°-60°, with pain beginning at 30° of flexion. Imaging revealed high-grade trochlear dysplasia (sulcus angle 166°), a tibial tubercle-to-trochlear groove (TT-TG) distance of 27 mm, and a Caton-Deschamps index (CDI) of 1.37 (Figures 3A-3D), consistent with patella alta and lateralized TT alignment.

Surgical correction of the long-standing patellar dislocation was undertaken, consisting of (1) trochleoplasty, (2) tibial tubercle osteotomy (TTO), (3) MPFL reconstruction, and (4) quadricepsplasty, which was considered optional and performed only if knee flexion remained restricted after completion of the other procedures.

The rationale for this multimodal approach was the coexistence of several anatomical abnormalities, and the surgical plan followed Dejour’s “menu à la carte” principles for patellar instability [8]. Trochleoplasty was indicated as the primary intervention due to severe trochlear dysplasia. TTO, with medialization and slight distalization, addressed extensor mechanism malalignment, given the TT-TG distance >20 mm and CDI >1.2. MPFL reconstruction, a systematic component of Dejour’s protocol, was included to restore medial patellar restraint and prevent recurrent lateral displacement.

**Figure 4:**
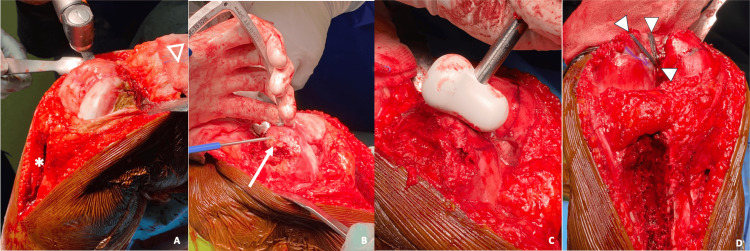
Intraoperative steps of trochleoplasty (A) Trochlear exposure after tibial tubercle osteotomy (asterisk) and proximal reflection of the extensor apparatus (hollow arrowhead); (B) mobilization of a 5-mm osteochondral flap (arrow) using a burr and an offset guide; (C) reshaping of the trochlea with sulcus deepening and removal of the trochlear bump; (D) final result after fixation with three anchors (arrowheads), showing the new morphology of the trochlear groove.

The operation began with TTO and release of both retinacula, allowing proximal reflection of the patella and exposure of the trochlea. Trochleoplasty was performed according to the Bereiter technique [9], a modification of Dejour’s method. A 3-5 mm osteochondral flap was mobilized with a high-speed burr and offset guide, the trochlear bump was resected, and the groove was deepened to increase lateral inclination (Figures 4A-4D). The TT was then fixed in the corrected position (≈5 mm medialized and slightly distalized), confirming proper patellofemoral tracking. The MPFL was reconstructed with the ipsilateral semitendinosus tendon to restore medial stability.

Despite these corrections, intraoperative flexion remained limited to approximately 60°, attributed to residual quadriceps tension from the previous lengthening procedure. A V-Y quadricepsplasty, mainly involving the vastus lateralis belly, was therefore added to release the contracture and improve flexion. At the end of the procedure, patellar tracking was stable, without dislocation or subluxation throughout 0°-100° of motion.

Postoperatively, the patient remained non-weight-bearing for six weeks due to the osteotomy, followed by gradual partial weight-bearing and physiotherapy, focusing on quadriceps flexibility and patellar tracking. At the six-month follow-up, he walked independently, with complete osteotomy consolidation and no recurrent instability.

At one-year follow-up, the range of motion was 0°-85°, representing a loss of approximately 15° of flexion compared with the immediate postoperative period in the operating room. Despite this, the patient reported a high level of satisfaction with the surgical outcome (8/10) and a substantial functional improvement compared with his preoperative status. He was able to perform daily activities without significant discomfort and could run short distances, though mild limitations persisted during squatting, prolonged walking, and sports activities. Functional evaluation using validated outcome measures showed Lysholm and Gillquist [10] and Kujala et al. [11] scores of 71 and 65, respectively, indicating a satisfactory, yet incomplete, recovery of knee function.

## Discussion

This case illustrates the complexity and potential consequences of femoral lengthening, as well as its interference with the extensor mechanism of the knee. The patient underwent four distinct surgical procedures: (1) lengthening surgery; (2) fracture fixation with correction of regenerate deformity; (3) removal of osteosynthesis material; and (4) correction of femoropatellar instability. Altogether, these procedures required seven years of follow-up at our institution, with multiple hospital admissions and direct consequences on the patient’s life between the ages of 15 and 22. The final outcome was a significant improvement in function and motor capacity, with correction of the initial discrepancy and restoration of the extensor mechanism alignment.

Complications of lengthening

Distraction osteogenesis, or bone lengthening, is well established in current practice; nevertheless, complication rates remain relatively high. In a study by Castelein and Docquier, among 51 lengthenings performed, 84% developed complications, most of them minor and related to pin-site infections. Major complications, such as fractures, axis deviation, non-union, or joint contractures, occurred in 10%-15% of patients [12]. Some studies report an even higher incidence of regenerate fractures, ranging from 3.6% to 50% [13]. Traditionally, bone lengthening has been performed with monoplane or circular external fixators. Between these devices, the monoplane offers practical advantages, such as a shorter operative time, simpler application, and better patient tolerance, particularly when long fixation periods are required, as in the correction of large discrepancies. However, monoplane constructs provide less axial and torsional stability, which likely explains the higher incidence of major complications (loss of alignment, axis deviation, fractures, and non-unions) compared with circular fixators [12]. This reduced stability can also compromise regeneration quality, especially in long distraction segments, possibly contributing to the fracture that occurred shortly after fixator removal.

Technological advances and mechanical considerations

More recently, modern systems, such as hexapod frames and intramedullary lengthening nails, have gained prominence. These devices offer improved stability, more accurate alignment control, shorter external fixation times, and greater patient comfort, resulting in lower complication rates and higher functional satisfaction compared with traditional external fixators [14]. At the time of the index surgery, however, magnetically controlled intramedullary systems were not yet available in our institution and remained cost-prohibitive, influencing the decision to proceed with a monoplane external fixator. The lengthening-over-nail technique, using a conventional intramedullary femoral nail combined with a monolateral external fixator, was already established and could have been considered as an alternative. In a matched comparative study, Paley et al. reported that this approach reduced the duration of external fixation by nearly 50%, accelerated regenerate consolidation, and improved recovery of knee motion, virtually eliminating refractures seen with conventional Ilizarov methods [15]. In retrospect, adopting this technique might have prevented the regenerate fracture in the present case. These insights illustrate the rapid technological evolution in limb-lengthening surgery and reinforce the importance of mechanical stability for long-term success.

Mechanism of instability

The patient subsequently developed an obligate patellar dislocation, a condition that typically arises from combined abnormalities of the trochlear morphology, MPFL, TT-TG relationship, and patellar height [7,16]. In this case, the dislocation was attributed to the cumulative effects of quadriceps contracture, severe trochlear dysplasia, increased TT-TG distance, and patella alta following femoral distraction osteogenesis. Intraoperatively, marked quadriceps tension and restricted knee flexion were observed, both of which improved only after V-Y lengthening. Similar findings have been described by Martin et al., who reported a subset of patients developing patellar instability secondary to excessive extensor mechanism tension after femoral lengthening. In their series, quadricepsplasty restored flexion and corrected obligate dislocation, with a mean improvement of knee motion from 48° to 120° and stable tracking at final follow-up [5].

Surgical strategy

The multimodal surgical approach, comprising trochleoplasty, TTO, MPFL reconstruction, and V-Y quadriceps lengthening, enabled correction of all contributing anatomic factors and restoration of stable patellar tracking. Following Dejour’s “menu à la carte” principle, each risk factor was addressed individually to achieve patellofemoral congruence while avoiding overcorrection or iatrogenic maltracking [8]. In this context, isolated MPFL reconstruction is suitable only for mild abnormalities, whereas more severe cases require additional procedures, such as TTO or trochleoplasty. The latter is typically reserved for high-grade trochlear dysplasia (types B, D, and some C) when the lateral trochlear inclination angle is <10°, aiming to deepen the sulcus and remove the trochlear bump [8,9,17,18]. Slightly increased femoral anteversion was not corrected, as the morbidity of an additional derotation osteotomy was deemed unnecessary, given the stability achieved with the selected interventions.

Outcomes and limitations

This case highlights the complex interplay between bony and soft-tissue factors in post-lengthening knee pathology and illustrates how sequential correction of each abnormality can restore stability and function. The clinical outcomes achieved at one year postoperatively demonstrate that, even in long-standing and multifactorial patellar instability, functional recovery is achievable when guided by a patient-specific, anatomy-based surgical plan.

This report is limited by its single-patient design and absence of postoperative gait or strength analysis. Nonetheless, at one year, the patient achieved satisfactory functional recovery, emphasizing that partial biomechanical restoration can yield significant symptomatic improvement.

Future perspectives

Although the use of artificial intelligence (AI) in limb-lengthening procedures remains largely unexplored, recent developments in AI-assisted imaging and predictive modeling suggest that these tools may, in the future, help anticipate and prevent mechanical or regenerative complications. Current studies have demonstrated that deep learning algorithms can accurately assess lower limb alignment and leg length discrepancies, achieving results comparable to expert radiologists [19]. In a broader context, AI is showing increasing potential for decision support, surgical planning, and postoperative monitoring across multiple medical and surgical specialties [20]. As these technologies continue to evolve, they may eventually enable more precise, personalized, and proactive management of patients undergoing distraction osteogenesis, although their adoption is still constrained by cost and availability in most orthopedic settings.

## Conclusions

This case highlights the importance of long-term biomechanical re-evaluation following femoral lengthening, as subtle residual deformities or extensor mechanism imbalance may lead to late complications, such as chronic patellar instability. Correction of the underlying abnormalities, including reduction of the TT-TG distance, normalization of patellar tracking, and recovery of a functional range of motion, resulted in a satisfactory clinical outcome and high patient satisfaction. This case also demonstrates that a tailored, combined surgical approach, addressing both bony and soft-tissue factors, can achieve stability and functional recovery even in long-standing, complex cases. 
